# Recurrent Primary Hyperparathyroidism Due to Adenoma Formation in Autotransplanted Parathyroid Tissue 28 Years After Parathyroidectomy in a Patient With Multiple Endocrine Neoplasia Type 2A

**DOI:** 10.7759/cureus.114014

**Published:** 2026-08-05

**Authors:** Roman Farnin, Robert Lienenlüke, Johanna Engler, Halil Altindag, Antonia Hammer, Lula Gebrehiwot, Julian Mittermeier, Annas Jomaa Zabadné, Andrea Sergio Merlo, Christian Vorländer

**Affiliations:** 1 Department of Endocrine Surgery, Bürgerhospital Frankfurt am Main, Frankfurt am Main, DEU

**Keywords:** 18f-choline pet/ct, multiple endocrine neoplasia type 2a, parathyroid adenoma, parathyroid autotransplantation, primary hyperparathyroidism, recurrent hyperparathyroidism, ret mutation

## Abstract

Primary hyperparathyroidism (PHPT) is an established manifestation of multiple endocrine neoplasia type 2A (MEN2A). In patients requiring extensive parathyroid surgery, autotransplantation of parathyroid tissue may be performed to preserve parathyroid function. Recurrence due to adenoma formation within autotransplanted parathyroid tissue is exceptionally rare.

We report a case of a 59-year-old male with genetically confirmed MEN2A caused by a heterozygous RET p.Cys634Tyr (C634Y) mutation who developed recurrent PHPT caused by adenoma formation within autotransplanted parathyroid tissue 28 years after parathyroid surgery with autotransplantation into the sternocleidomastoid muscle. Biochemical evaluation demonstrated hypercalcemia and elevated parathyroid hormone (PTH) levels. Fluorine-18 choline (¹⁸F-choline) positron emission tomography/computed tomography (PET/CT) localized a hyperfunctioning lesion within the autotransplanted parathyroid tissue. Surgical excision resulted in normalization of biochemical parameters, and histopathological examination confirmed a parathyroid adenoma.

This case demonstrates that autotransplanted parathyroid tissue may remain capable of adenoma formation decades after transplantation in patients with MEN2A. It underscores the importance of lifelong biochemical surveillance and highlights the value of ¹⁸F-choline PET/CT for accurate localization of recurrent hyperfunctioning autotransplanted parathyroid tissue.

## Introduction

Multiple endocrine neoplasia type 2A (MEN2A) is an autosomal dominant syndrome caused by germline activating mutations of the RET proto-oncogene and characterized by a predisposition to medullary thyroid carcinoma (MTC), pheochromocytoma, and primary hyperparathyroidism (PHPT) [1,2]. PHPT is the least common component of MEN2A and develops in a minority of affected individuals, with the highest penetrance observed among carriers of RET codon 634 mutations [3,4]. Although historically reported in up to 20-30% of patients, recent population-based studies suggest a lower prevalence of approximately 8-11% [3,4]. PHPT is typically the last MEN2A manifestation to emerge clinically, with a median age at diagnosis of approximately 39-45 years, highlighting the importance of lifelong surveillance in RET mutation carriers [5,6].

Surgical management of PHPT in MEN2A includes selective resection of enlarged glands, subtotal parathyroidectomy, or total parathyroidectomy with autotransplantation, depending on the extent of parathyroid involvement. Parathyroid autotransplantation involves implantation of viable parathyroid tissue into skeletal muscle to preserve parathyroid function and reduce the risk of permanent hypoparathyroidism after extensive parathyroid resection or when the viability of a parathyroid gland is compromised. Following successful engraftment, the transplanted tissue is expected to remain functional and provide long-term parathyroid hormone (PTH) secretion [5,7,8]. Although recurrent hyperparathyroidism may arise from residual cervical tissue or autotransplanted glands, recurrence originating from autotransplanted parathyroid tissue appears to be uncommon in MEN2A patients [5,9]. Hyperparathyroidism has been reported even in autografts derived from histologically normal parathyroid tissue; recurrent disease may be associated with hyperplastic proliferation of the graft or with the development of a discrete parathyroid adenoma [10].

Only a few cases of recurrent PHPT arising within autotransplanted parathyroid tissue in MEN2A have been reported, and latency periods exceeding two decades are exceptionally rare [11,12]. We report a patient with genetically confirmed MEN2A caused by a heterozygous RET p.Cys634Tyr (C634Y) mutation who developed recurrent PHPT due to a histologically confirmed parathyroid adenoma arising in autotransplanted parathyroid tissue within the sternocleidomastoid muscle 28 years after the initial surgery. In addition to the exceptionally long latency period, this case illustrates the diagnostic challenge of distinguishing a hyperfunctioning parathyroid autograft lesion from nodal recurrence of MTC in a previously operated patient with MEN2A and highlights the value of functional imaging for accurate lesion localization. The exceptionally late recurrence further underscores the importance of lifelong surveillance in MEN2A patients.

## Case presentation

A 59-year-old male with genetically confirmed MEN2A caused by a heterozygous RET p.Cys634Tyr (C634Y) mutation was referred for evaluation of recurrent PHPT. The family history was consistent with hereditary MEN2A involving multiple generations. His medical history was notable for bilateral pheochromocytomas, MTC, PHPT, and parathyroid autotransplantation. A summary of the clinical course is provided in Table 1.

**Table 1:** Timeline of major clinical events MEN2A: multiple endocrine neoplasia type 2A; TNM: tumor, node, and metastasis.

Year	Clinical event
1988	Left nephrectomy with ipsilateral adrenalectomy for a large left renal mass; pheochromocytoma was recognized only later in the clinical course.
1995	Right adrenalectomy for pheochromocytoma.
1997	Diagnosis of MEN2A.
1998	Total thyroidectomy, bilateral neck dissection, transcervical thymectomy, excision of a right superior parathyroid adenoma, and autotransplantation of the right inferior parathyroid gland into the right sternocleidomastoid muscle (TNM pT1b pN1b (9/33) M0 R0).
1999	Reoperation for recurrent left-sided pheochromocytoma.
2024-2026	Progressive symptoms and biochemical findings consistent with recurrent primary hyperparathyroidism.
2026	Excision of a parathyroid adenoma arising within the autotransplanted parathyroid graft.

In 1998, the patient underwent total thyroidectomy, bilateral modified radical neck dissection, transcervical thymectomy, excision of a right superior parathyroid adenoma, and autotransplantation of the right inferior parathyroid gland into the ipsilateral sternocleidomastoid muscle. Frozen-section examination confirmed a right superior parathyroid adenoma, while the autotransplanted gland was free of adenomatous changes. Final histopathology demonstrated multifocal MTC with C-cell hyperplasia and cervical lymph node metastases. Following the 1998 surgery, serum calcium and PTH levels were monitored annually as part of long-term follow-up.

The patient remained under regular endocrinological surveillance for many years. Beginning in 2022, serial biochemical measurements demonstrated a gradual increase in PTH levels, followed by the development of hypercalcemia in 2023. During the subsequent two years, he developed progressive symptoms consistent with recurrent PHPT, including fatigue, depression, osteoporosis, and musculoskeletal pain involving the shoulders and knees. Overt biochemical recurrence became evident in May 2024, with a serum calcium level of 2.80 mmol/L (reference range: 2.1-2.65 mmol/L) and a PTH concentration of 113 pg/mL (reference range: 4-85 pg/mL). Serum 25-hydroxyvitamin D was 30 ng/mL in 2022 (reference range: 30-100 ng/mL) and decreased to 15 ng/mL in 2024. Despite a history of MTC, serum calcitonin levels remained low to only minimally elevated during follow-up, without clear biochemical evidence of recurrent MTC.

**Figure 1:**
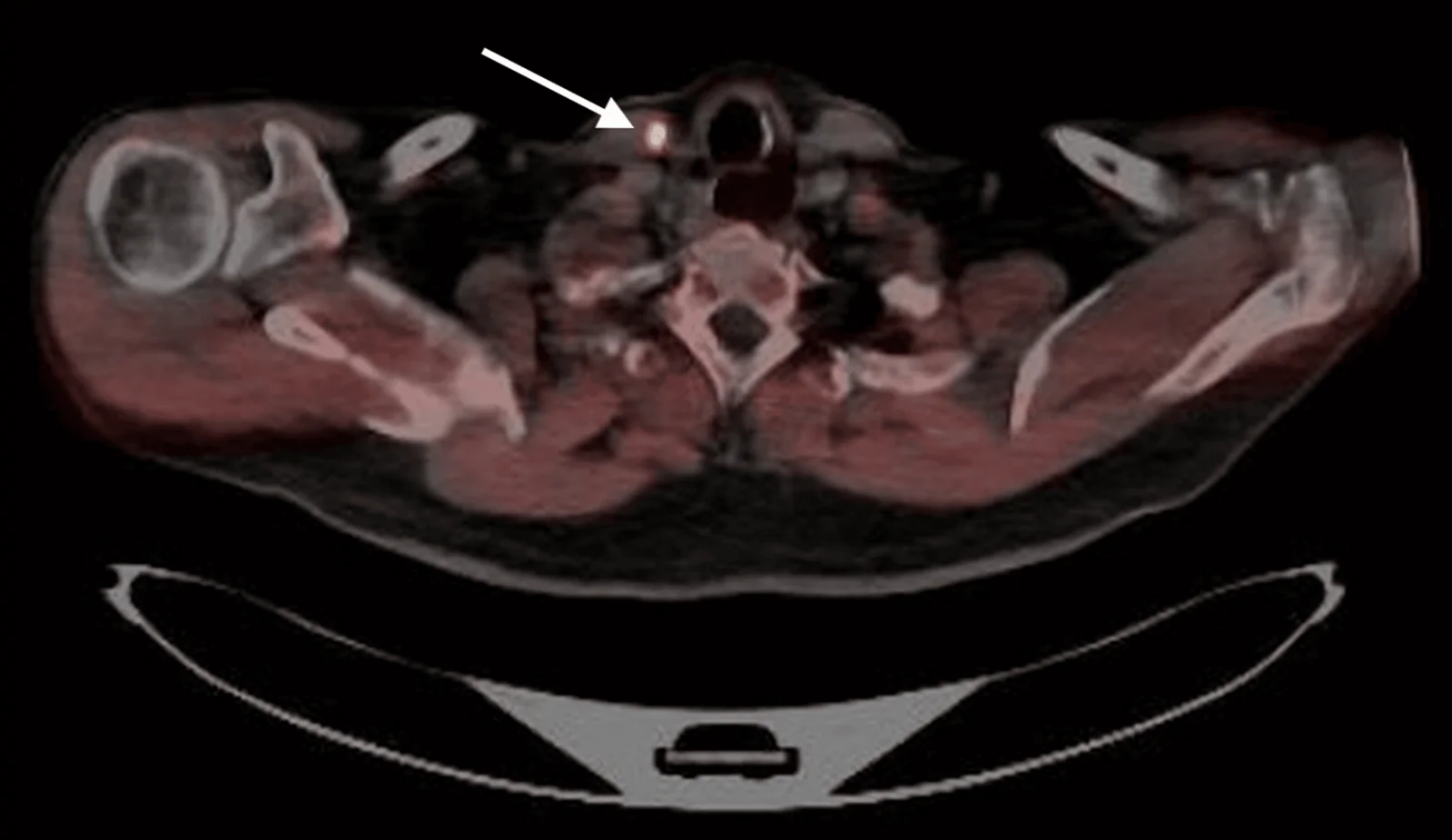
¹⁸F-choline PET/CT demonstrating focal tracer uptake within the autotransplanted parathyroid tissue. Axial ¹⁸F-choline PET/CT image demonstrating focal tracer uptake within the right sternocleidomastoid muscle at the site of previous parathyroid autotransplantation (arrow), consistent with a parathyroid adenoma arising within the autotransplanted parathyroid tissue.

Initial imaging included neck ultrasonography and technetium-99m (99mTc) sestamibi scintigraphy, which demonstrated a small right-sided cervical lesion that was initially interpreted as a suspicious lymph node in the setting of suspected recurrent MTC. A subsequent gallium-68 fibroblast activation protein inhibitor (⁶⁸Ga-FAPI) positron emission tomography/computed tomography (PET/CT) performed to evaluate possible recurrent MTC was non-diagnostic with respect to the source of hyperparathyroidism. Finally, fluorine-18 choline (¹⁸F-choline) PET/CT demonstrated focal tracer uptake within the right sternocleidomastoid muscle at the site of previous parathyroid autotransplantation, with a maximum standardized uptake value (SUVmax) of 12.6 (Figure 1), prompting targeted ultrasonographic reassessment and confirmation of the lesion.

**Figure 2:**
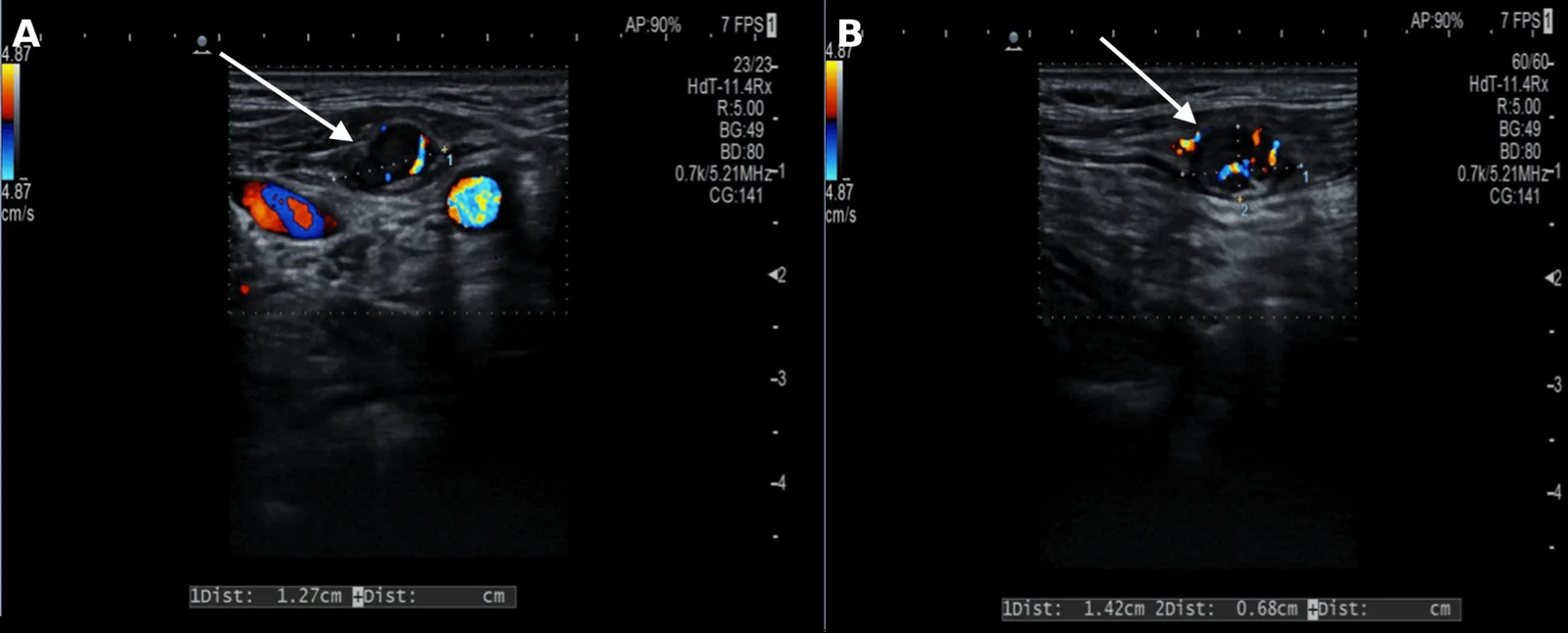
Targeted ultrasonographic reassessment of the autotransplantation site following localization by ¹⁸F-choline PET/CT. (A) Transverse color Doppler image demonstrating a well-circumscribed hypoechoic hypervascular lesion within the right sternocleidomastoid muscle. (B) Longitudinal view of the same lesion measuring 1.42 × 0.68 cm, corresponding to the hyperfunctioning lesion identified on ¹⁸F-choline PET/CT.

Following localization by ¹⁸F-choline PET/CT, targeted ultrasonographic reassessment of the previous autotransplantation site demonstrated a well-circumscribed hypoechoic hypervascular lesion within the right sternocleidomastoid muscle, measuring 1.42 × 0.68 cm (Figure 2).

**Figure 3:**
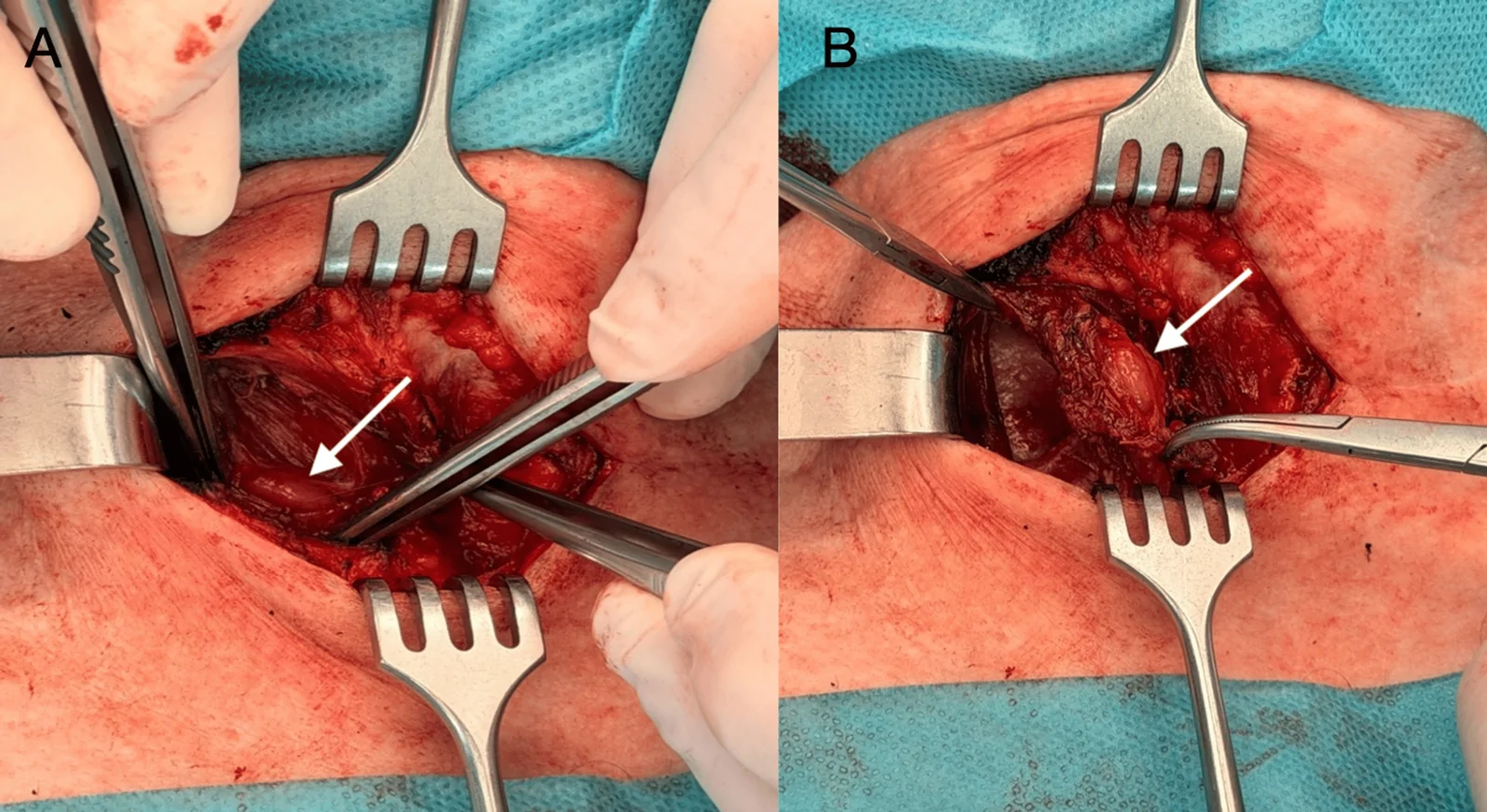
Intraoperative excision of the parathyroid adenoma arising within autotransplanted parathyroid tissue. (A) Intraoperative exposure of a well-circumscribed lesion within the right sternocleidomastoid muscle at the site of previous parathyroid autotransplantation. (B) Mobilization and complete excision of the parathyroid adenoma arising within the autotransplanted parathyroid tissue. Subsequent histopathological examination confirmed a parathyroid adenoma, with no evidence of metastatic medullary thyroid carcinoma.

Based on the biochemical evidence of recurrent PHPT and concordant localization by ¹⁸F-choline PET/CT and targeted ultrasonography, focused surgical exploration was recommended. In June 2026, the patient underwent excision of the lesion located within the right sternocleidomastoid muscle at the site of the previous parathyroid autotransplantation. Intraoperatively, a well-circumscribed nodular lesion was identified within the sternocleidomastoid muscle and completely excised (Figure 3). During the early postoperative period, serum PTH levels decreased from 167 pg/mL before surgery to 12 pg/mL, consistent with successful removal of the hyperfunctioning parathyroid tissue. Given the uncertainty regarding the presence and functional status of the remaining left-sided parathyroid glands following the extensive cervical surgery performed in 1998, a portion of the resected parathyroid tissue was cryopreserved as a precautionary measure to preserve the option of future autotransplantation in the event of permanent hypoparathyroidism. Continued long-term biochemical and clinical follow-up is planned, with future use of the cryopreserved tissue to be considered only if clinically indicated.

**Figure 4:**
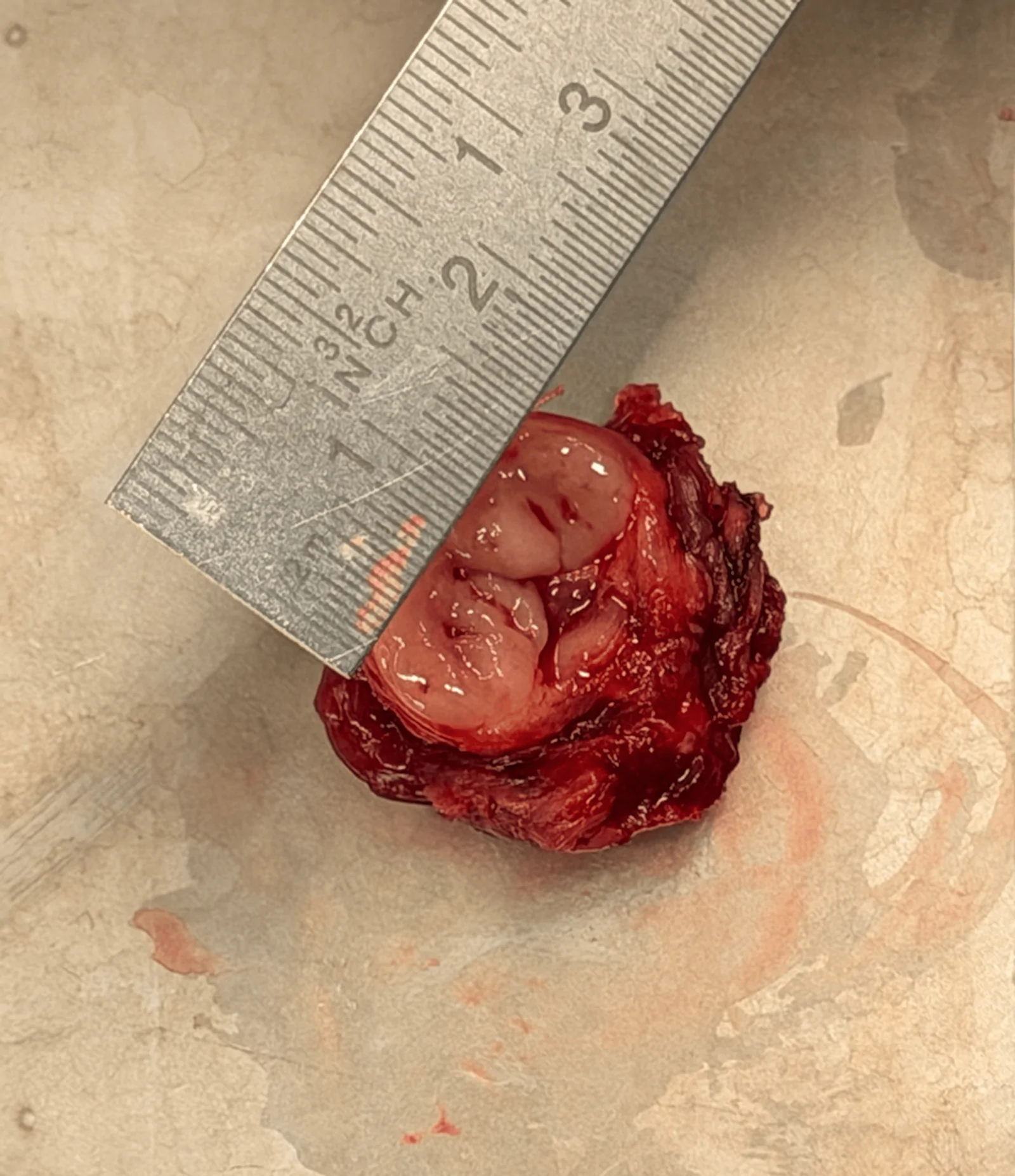
Gross specimen of the excised parathyroid adenoma. Sectioned specimen showing a well-circumscribed nodular lesion with a homogeneous tan cut surface, subsequently confirmed histologically as a parathyroid adenoma, with no evidence of metastatic medullary thyroid carcinoma. A ruler is included for size reference.

Gross examination of the resected specimen revealed a well-circumscribed nodular lesion with a homogeneous cut surface (Figure 4). Histopathological examination confirmed a parathyroid adenoma arising within the autotransplanted parathyroid tissue. No evidence of recurrent MTC was identified.

The postoperative course was uneventful. At hospital discharge, serum calcium was 2.17 mmol/L (reference range: 2.2-2.65 mmol/L), and PTH was 28 pg/mL (reference range: 15-68 pg/mL), consistent with biochemical remission. The patient was discharged in good clinical condition and remains under regular endocrinological surveillance.

## Discussion

This case represents a very late recurrence of PHPT caused by adenoma formation within autotransplanted parathyroid tissue, occurring 28 years after the initial transplantation. The prolonged latency period highlights the potential for autonomous growth of autotransplanted parathyroid tissue even decades after surgery and underscores the importance of lifelong biochemical surveillance in patients with MEN2A, particularly those carrying RET codon 634 mutations.

PHPT is the least common manifestation of MEN2A, with the highest penetrance reported among carriers of RET codon 634 mutations [5,13]. In contrast to sporadic PHPT and MEN1-associated disease, MEN2A-associated hyperparathyroidism is generally characterized by a milder clinical phenotype, lower biochemical activity, and a later age of presentation [5,14]. Consequently, surgical management remains controversial. Current American Thyroid Association and American Association of Endocrine Surgeons guidelines favor selective resection of visibly enlarged glands, whereas subtotal or total parathyroidectomy with autotransplantation is generally reserved for patients with multiglandular disease. Each approach represents a balance between the risks of recurrent hyperparathyroidism and permanent hypoparathyroidism [5,15].

The most remarkable aspect of the present case is the development of a parathyroid adenoma within autotransplanted parathyroid tissue nearly three decades after transplantation. At the time of the initial surgery in 1998, frozen-section examination confirmed adenomatous involvement of the right superior parathyroid gland, whereas the right inferior gland selected for autotransplantation showed no evidence of adenomatous transformation and was considered histologically normal. Nevertheless, recurrent PHPT subsequently developed due to adenoma formation within the autograft. Although the exact mechanism remains unclear, possible explanations include de novo adenomatous transformation of transplanted parathyroid tissue or gradual clonal expansion of cells with latent proliferative potential. Further molecular characterization of the resected adenoma could provide additional insight into the biological mechanisms underlying very late autograft recurrence, although distinguishing de novo adenomatous transformation from clonal expansion of a pre-existing cell population would remain challenging without comparative archival tissue from the original surgery. These observations suggest that autotransplanted parathyroid tissue may retain the capacity for autonomous growth many years after transplantation [11,12].

Only a few cases of recurrent PHPT arising within autotransplanted parathyroid tissue in MEN2A have been reported. One previously described case involved hyperplastic transformation of the autograft 18 years after transplantation [11], whereas another documented recurrent disease originating from autotransplanted tissue [12]. In the present case, a histologically confirmed parathyroid adenoma developed within the autotransplanted parathyroid tissue 28 years after transplantation. To our knowledge, this represents one of the longest reported latency periods between parathyroid autotransplantation and clinically significant recurrent PHPT in a patient with MEN2A.

Alternative contributors to the elevated PTH level were also considered. Serum 25-hydroxyvitamin D decreased from 30 ng/mL in 2022 to 15 ng/mL in 2024 and may therefore have contributed to the degree of PTH elevation. However, the concomitant hypercalcemia, localization of a hyperfunctioning lesion at the documented autotransplantation site, histological confirmation of a parathyroid adenoma, and postoperative normalization of PTH with resolution of hypercalcemia supported recurrent PHPT arising from the autograft as the principal cause. Occult residual cervical parathyroid tissue was considered less likely given the lesion localization and postoperative biochemical response.

An additional diagnostic challenge in the present case was the patient's history of MTC. A retrospective review demonstrated that the lesion had been identified on both ultrasonography and 99mTc-sestamibi scintigraphy before ¹⁸F-choline PET/CT was performed. However, because of concern for recurrent MTC, the finding was initially interpreted as a suspicious cervical lymph node rather than recurrent hyperfunctioning autotransplanted parathyroid tissue. In retrospect, awareness of the documented parathyroid autotransplantation into the right sternocleidomastoid muscle could have broadened the differential diagnosis of the cervical lesion to include hyperfunctioning autotransplanted parathyroid tissue. This observation highlights the potential value of reviewing previous operative records in patients with complex endocrine surgical histories. Serial biochemical measurements demonstrated a gradual increase in PTH levels over several years before the diagnosis was established. Although the lesion was retrospectively identifiable on conventional imaging, its interpretation was complicated by the clinical context of MEN2A and previous MTC. A subsequent ⁶⁸Ga-FAPI PET/CT examination performed to evaluate possible recurrent MTC was non-diagnostic with respect to the source of hyperparathyroidism. In contrast, ¹⁸F-choline PET/CT successfully localized a hyperfunctioning lesion within the right sternocleidomastoid muscle at the site of previous parathyroid autotransplantation, demonstrating increased tracer uptake with an SUVmax of 12.6 and allowing targeted ultrasonographic reassessment and focused surgical excision. As the lesion was accessible by ultrasonography, ultrasound-guided fine-needle aspiration with PTH measurement in the needle washout could also have been considered as an alternative diagnostic approach. A markedly elevated PTH concentration in the needle washout could have supported a parathyroid origin of the lesion and helped distinguish hyperfunctioning autotransplanted parathyroid tissue from recurrent MTC before proceeding to ¹⁸F-choline PET/CT [16]. Together, these findings highlight the diagnostic challenges associated with recurrent hyperparathyroidism arising within autotransplanted tissue and support the growing role of ¹⁸F-choline PET/CT in the evaluation of persistent or recurrent hyperparathyroidism, particularly in previously operated necks.

## Conclusions

This case demonstrates that recurrent PHPT may arise from autotransplanted parathyroid tissue even decades after apparently successful surgery in patients with MEN2A. The development of a histologically confirmed parathyroid adenoma within autotransplanted parathyroid tissue 28 years after transplantation highlights the potential for very late recurrence and supports the need for lifelong biochemical surveillance in this population. Furthermore, this case illustrates the diagnostic challenges associated with recurrent hyperparathyroidism in previously operated patients with MEN2A and a history of medullary thyroid carcinoma, and emphasizes the value of ¹⁸F-choline PET/CT for accurate localization of hyperfunctioning autotransplanted parathyroid tissue. As this report describes a single patient, these observations should not be interpreted as generalizable treatment recommendations but may support consideration of long-term surveillance and advanced functional imaging in comparable clinical settings.
